# Immune Biomarkers of Islet Transplant Rejection Revealed by Synthetic Immunological Niche

**DOI:** 10.1002/advs.78154

**Published:** 2026-10-08

**Authors:** Jyotirmoy Roy, Abdalmonam Jadou Nejma, Mohammad Tarique, Amod Talekar, Shengli Wu, Brianna Ha, Yifei Jiang, Esma S. Yolcu, Lonnie D. Shea

**Affiliations:** ^1^ Department of Biomedical Engineering University of Michigan Ann Arbor USA; ^2^ Department of Pediatrics Department of Molecular Microbiology and Immunology NextGen Precision Health Institute University of Missouri Columbia USA; ^3^ Department of Chemical Engineering University of Michigan Ann Arbor USA

**Keywords:** biopsy, early rejection biomarker, immune dysregulation, immunotherapy, type 1 diabetes

## Abstract

Islet transplantation can restore glycemic control in type 1 diabetes, yet heterogeneity of immune responses and transplant outcomes necessitates improved graft monitoring. Since transplanted islets are diffusely engrafted within the liver and inaccessible to biopsy, clinical monitoring relies on blood glucose and C‐peptide, which change only after substantial graft injury. Here, we developed a synthetic immunological niche (IN) that captures graft‐associated immune responses through serial subcutaneous biopsy. We evaluated the IN across murine syngeneic, allogeneic, and autoimmune islet transplant models, including CD40/CD154 blockade with anti‐CD40L. The IN identified immune populations and transcriptomic signatures reflective of the graft and distinguished syngeneic from rejecting allografts. In anti‐CD40L‐treated allografts, the IN revealed innate immune programs characterized by macrophage‐ and dendritic cell‐associated signatures linked to acceptance versus rejection, whereas untreated allografts showed stronger adaptive immune signatures. Longitudinal IN profiling detected inflammatory activation in accepted allografts, indicating persistent subclinical risk. Finally, in autoimmune allografts treated with anti‐CD40L plus rapamycin, IN identified a 13‐gene signature that separated early from late rejection and distinguished autoimmune‐ from alloimmune‐associated rejection programs. These findings establish the IN as a platform for identification of early biomarkers of islet graft rejection before overt dysfunction, addressing an unmet need in islet transplant monitoring.

## Graphical abstract

A subcutaneous synthetic immunological niche (IN) enables serial, minimally invasive monitoring of immune responses after islet transplantation. Flow cytometry and transcriptomic profiling of the IN reveal immune‐cell programs associated with graft acceptance and distinct types of rejection. Importantly, IN‐derived immune signatures indicate graft rejection before blood glucose rises, addressing an unmet need in islet transplant monitoring.

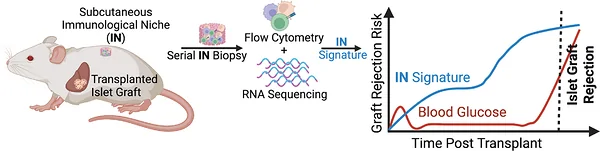

## 1 Introduction

Islet transplantation offers a transformative treatment option for individuals with brittle autoimmune type 1 diabetes (T1D), characterized by marked glycemic variability and recurrent episodes of severe, life‐threatening hypoglycemia [1, 2, 3]. Islet transplants restore endogenous insulin secretion, thereby improving glycemic stability and reducing the risk of severe hypoglycemia [4, 5, 6, 7, 8]. Most clinical islet transplants are heavily dependent on some form of immunosuppression to control rejection and recurrent autoimmunity. However, clinical outcomes are still highly heterogeneous, with only 37.5% of recipients achieving insulin independence at 1 year, 57%–77% maintaining HbA1c <7% long‐term, and 69%–84% retaining graft function during 3–8 years of follow‐up [9, 10, 11]. The variability arises primarily because durable graft function remains limited by heterogeneity in immune‐mediated loss of transplanted islets [12]. Unlike solid‐organ transplantation such as kidney, liver, and heart, where biopsies, imaging, and better‐defined circulating biomarkers can aid clinical surveillance, monitoring of islet graft health remains uniquely challenging [13, 14, 15, 16]. Transplanted islets are dispersed, inaccessible to routine biopsy, and have a relatively low tissue volume [17]. Changes in blood glucose or C‐peptide often become evident only after substantial graft injury has already occurred, leaving a narrow or absent window for timely therapeutic intervention and graft salvage [18, 19]. The delayed clinical detection of rejection and reliance on late functional readouts underscore the need for early biomarkers of immune dysregulation associated with islet graft rejection.

This challenge of monitoring tissue‐associated immune dysregulation during disease pathogenesis has been previously addressed with a microporous polycaprolactone scaffold that forms a synthetic immunological niche (IN) following subcutaneous implantation [20, 21]. The IN recruits host immune and stromal cells, through a foreign body response, to generate a vascularized, accessible microenvironment that mirrors systemic and tissue‐level immune states, while serving as a non‐vital tissue for serial biopsy. Prior studies demonstrated that longitudinal IN biopsy enables monitoring of dynamic immune programs across diverse pathological settings, especially autoimmune diseases and organ transplantation, without altering disease progression or transplant outcomes [20, 22, 23]. In T1D, longitudinal profiling of IN in non‐obese diabetic (NOD) mice enabled identification of early myeloid‐associated immune dysregulation followed by lymphoid dysregulation at later stages of disease progression, revealing inflammatory signatures that precede overt hyperglycemia [24]. The IN also predicted responses to anti‐TNFα therapy in these NOD mice by capturing baseline immune state differences at the time treatment was initiated. These findings further demonstrated the ability of IN to capture critical tissue‐associated transcriptomic changes in immune cells and, in turn, monitor the efficacy of immunotherapies. The utility of the IN was further supported in models of heart and skin transplantation, where it detected immune signatures associated with acute cellular allograft rejection [22]. These signatures captured immune activation that preceded overt graft injury and rejection. Collectively, these studies highlight the advantages of IN biopsy over peripheral blood monitoring, as circulating immune cells are often relatively naive and resting, whereas tissue‐resident and tissue‐recruited immune cells are more activated, functionally conditioned, and therefore more reflective of ongoing disease processes [25, 26]. Accordingly, the ability of the IN to capture tissue‐associated immune responses provides a unique opportunity to monitor graft‐associated immune dysregulation during islet transplantation, where direct assessment of graft‐infiltrating immune cells is not clinically feasible.

In this report, we investigated the ability of IN to identify early molecular biomarkers of islet graft rejection across clinically relevant transplant settings, including anti‐CD40L immunotherapy and recurrent autoimmunity. Tegoprubart, an anti‐CD40L monoclonal antibody recently evaluated in clinical studies, has demonstrated promising outcomes in islet transplantation, supporting the translational relevance of CD40‐CD154 pathway blockade [27]. The utility of the IN was first assessed in syngeneic and allogeneic islet transplant models, with INs implanted subcutaneously at the time of transplantation. The IN captured innate and adaptive immune cell populations relevant to islet rejection and identified a gene signature distinguishing syngeneic from allogeneic recipients that paralleled many of the immune changes within the graft, supporting its use as a minimally invasive cell‐capture platform for graft monitoring. We next profiled IN biopsies from accepted and rejected allografts under anti‐CD40L treatment. Transcriptomic and flow cytometric analyses revealed macrophage‐ and dendritic cell‐ associated populations linked to graft acceptance relative to rejection. INs further demonstrated distinct rejection mechanisms, with anti‐CD40L‐treated allografts exhibiting predominantly innate immune‐associated rejection programs, in contrast to untreated allografts, which showed stronger adaptive immune signatures. INs also captured increasing immune‐inflammatory signals over time in accepted allografts relative to syngeneic controls, suggesting ongoing risk for later graft failure. Finally, in an autoimmune‐allogeneic transplant model treated with anti‐CD40L and rapamycin, IN identified a 13‐gene signature that distinguished early from late rejection trajectories. These analyses further suggested that adaptive immune programs were associated with autoimmunity, whereas innate immune programs were more strongly linked to alloimmune responses under anti‐CD40L. Together, these findings demonstrate that the minimally invasive IN platform can capture otherwise inaccessible graft‐associated immune dysregulation, offering the potential for earlier therapeutic intervention and improved long‐term preservation of islet graft function in clinical transplantation.

## 2 Results

### 2.1 IN Captures Differences Between Allogeneic and Syngeneic Immune Responses that are Mirrored in Islet Grafts

We applied the IN in an islet transplant model to capture immune differences between rejecting allogeneic and stable syngeneic grafts. Diabetes was induced in C57BL/6 mice using streptozotocin, followed by transplantation of either allogeneic islets (BALB/c to C57BL/6) or syngeneic islets (C57BL/6 to C57BL/6). INs were implanted subcutaneously at the time of transplant and serially biopsied with replacement from days 7–70 or until onset of hyperglycemia (Figure 1a). Allogeneic recipients without immunosuppression showed an initial reduction in blood glucose levels following transplantation; however, all mice subsequently rejected the engrafted islet cells and returned to overt hyperglycemia (≥250 mg/dL) by day 14 (Figure 1b,c and Table S1). In contrast, syngeneic recipients maintained normoglycemia through day 70, indicating durable graft function (Figure 1b,c). We next examined IN immune composition in the two groups at day 14 using spectral flow cytometry (Figure S1 and Table S2). Relative to syngeneic controls, allogeneic INs contained a greater proportion of total immune cells, consistent with heightened immune recruitment during graft rejection (Figure 1d). In the immune cell compartment, reduced neutrophil frequencies and a trend toward increased macrophage abundance in the allogeneic group were observed, with some cohort‐associated variability in the macrophages (Figure 1e,f). Dendritic cell proportions were similar among both groups (Figure 1g). Although total T cell proportions were comparable, allogeneic INs showed enrichment of CD4 T cells within the T cell compartment, whereas CD8 T cell frequencies were unchanged (Figure 1h–j), supporting a stronger helper/effector adaptive response in the IN during rejection. Syngeneic INs had an increase in CD43+CD8+ T cells, while CD43+CD4+ T cells were unchanged (Figure 1k,l). Since allogeneic grafts had developed hyperglycemia by day 14, this difference may reflect contraction or redistribution of activated CD8 T cells after peak graft injury. Syngeneic INs also showed higher proportions of total B cells and CD43+ B cells, consistent with recent studies highlighting a potential regulatory role of these B cell phenotypes [28] (Figure 1m,n). Most of these lymphoid subset populations were a small fraction of total immune cells. To ensure the IN captures transplanted graft‐associated immune responses, we performed a paired correlation analysis between the immune populations at the IN and liver graft‐site immune composition from the same animals at the endpoints (Figure 1o–t). In the allogeneic group, the IN had strong positive correlations with the liver at the endpoints (days 13–28) for total immune cell proportions and several innate immune populations, including neutrophils, macrophages, and dendritic cells (Figure 1o–r and Table S3). Associations for T and B cells also had a positive correlation, albeit less strong than innate immune cells and not reaching statistical significance (Figure 1s,t). In contrast, correlations were weaker in syngeneic recipients, likely because durable graft function and a stable immune environment at the endpoints (days 70–79) caused limited changes in the IN and graft immune profiles, reducing correlations between the two tissues. Together, these findings demonstrate that the IN recapitulates graft rejection‐associated immune features, supporting its use as a minimally invasive site for longitudinal monitoring of graft rejection.

FIGURE 1IN profiling distinguishes allogeneic and syngeneic islet transplants and reflects graft‐associated immune signatures. (a) Experimental design: Streptozotocin‐induced diabetic C57BL/6J mice received allogeneic (BALB/c) or syngeneic (C57BL/6J) islet transplants with concurrent subcutaneous IN implantation. INs were longitudinally biopsied with replacement until day 70 post‐transplant or onset of hyperglycemia. (b) Blood glucose trajectories, and (c) Diabetes‐free survival demonstrating rejection of allogeneic grafts (n = 10) by day 14, whereas syngeneic grafts (n = 7) remained normoglycemic through day 70. Proportions of (d) immune cells, (e) neutrophils, (f) macrophages, (g) dendritic cells, (h) T cells, (i) CD4+ T cells, (j) CD8+ T cells, (k) CD43+ CD4 T cells, (l) CD43+ CD8+ T cells, (m) B cells, and (n) CD43+ B cells in allogeneic (n = 8) and syngeneic (n = 7) INs at day 14. Data are shown as mean ± SEM. Statistical comparisons used unpaired t‐tests or Mann‐Whitney tests based on normality. (o‐t) Paired Pearson correlation analysis of immune population frequencies between the IN and liver graft site from the same animals at study endpoints, calculated separately for syngeneic (n = 7) and allogeneic (n = 10) recipients. Shaded regions represent 95% confidence intervals around the linear regression lines. Pearson's correlation coefficient (r) and two‐sided *p*‐values are shown. (u) Heatmap and (v) PCA showing elastic net‐derived IN transcriptomic signature separating allogeneic and syngeneic samples (n = 4–5/group per time point) across days 7 and 14. Ellipses represent 70% confidence intervals. (w) Cell type and (x) condition distribution from day 7 islet graft single‐cell RNA sequencing. (y) IN‐derived signature scores across graft cell types. (z) GSEA highlighting selected pathways enriched between allogeneic and syngeneic conditions in the IN.

**Figure:**
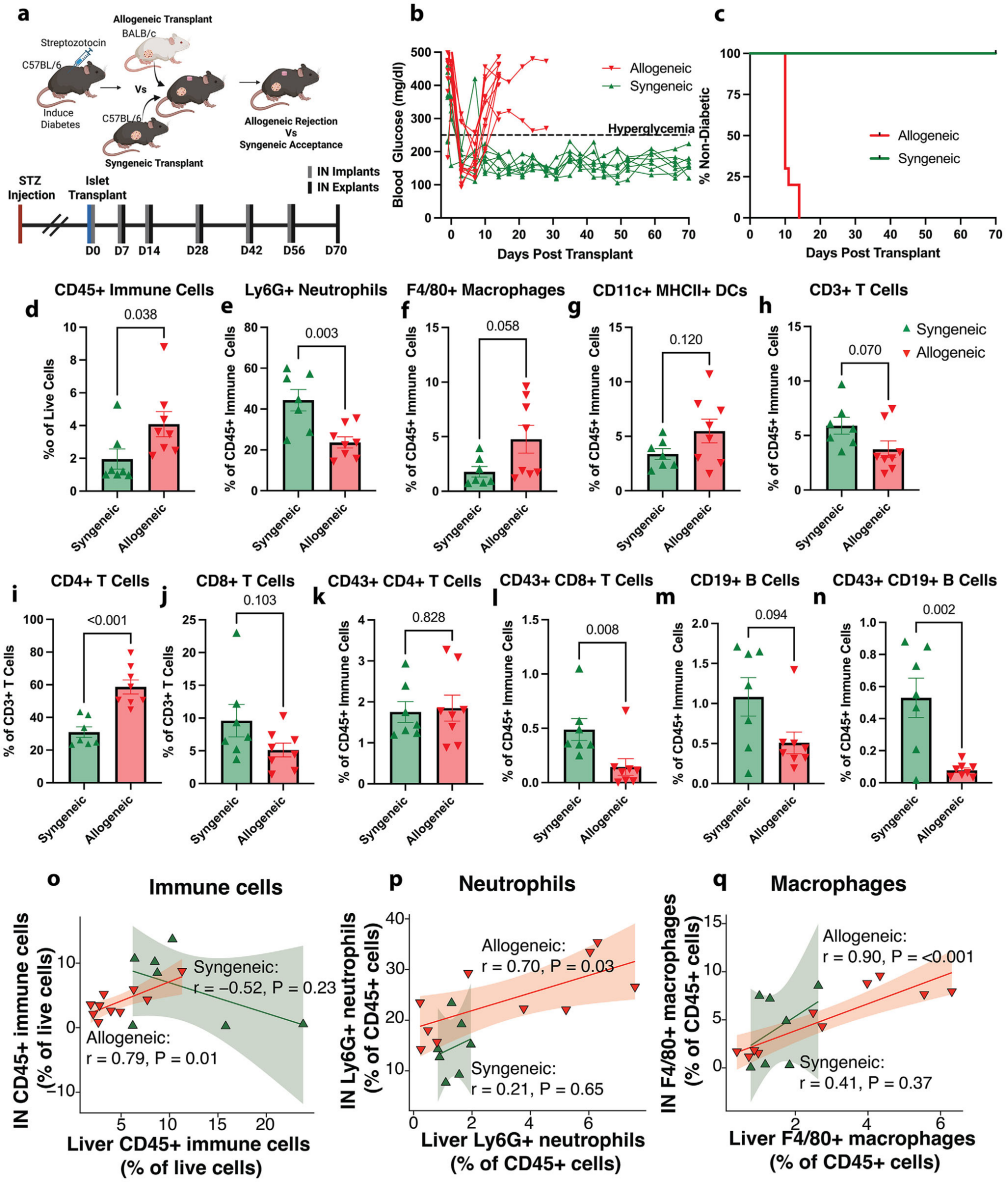

**Figure:**
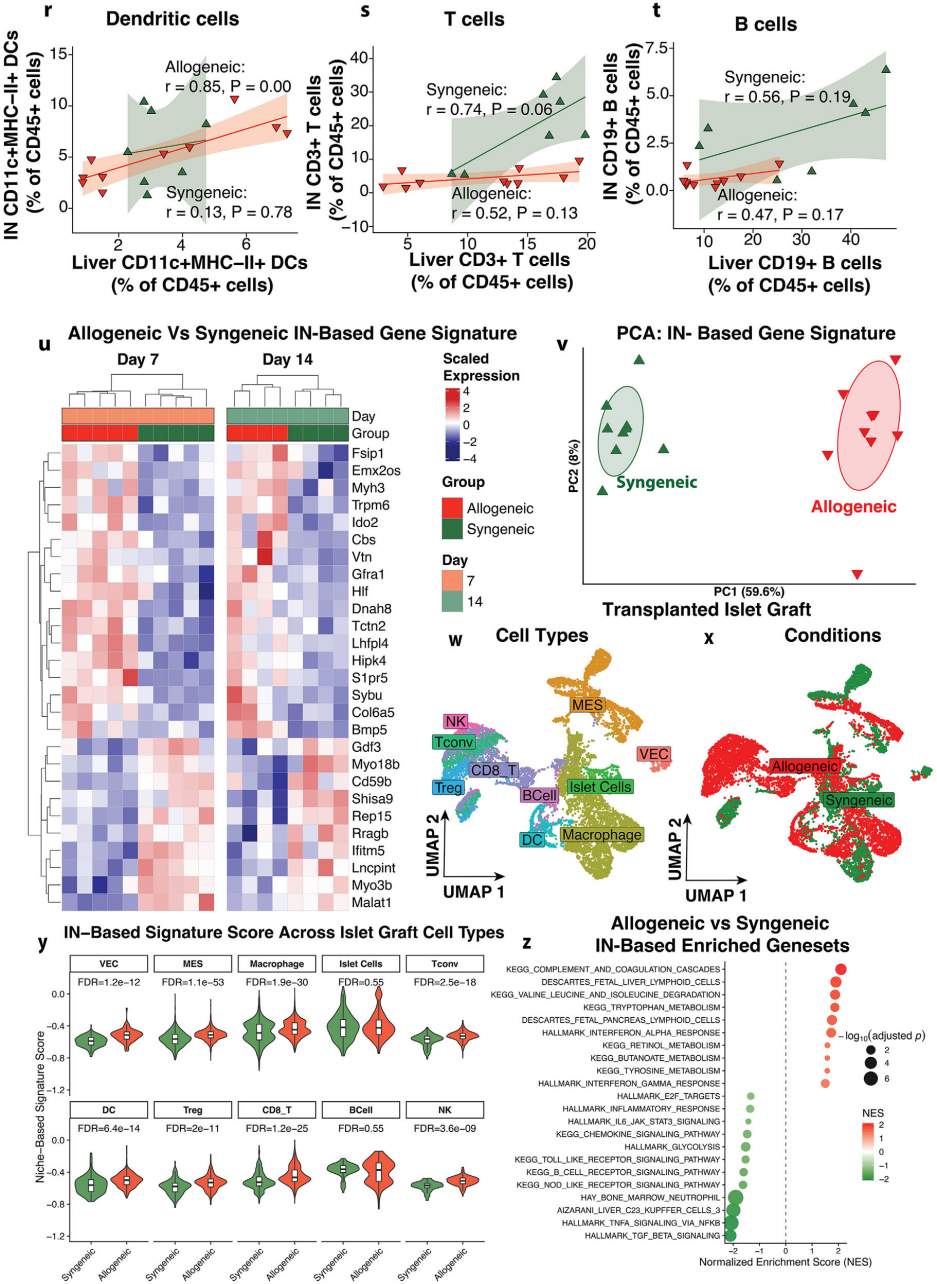

We next examined in‐depth phenotypic differences within IN using bulk RNA sequencing of IN biopsies collected at days 7 and 14 post‐transplant. After preprocessing and filtering, feature selection using an elastic net model (α = 0.5) identified a 27‐gene signature that robustly distinguished allogeneic from syngeneic recipients at both days 7 and 14, with the overall direction of the genes largely conserved across time (Figure 1u and Figure S2). Genes enriched in the syngeneic group included *Cd59b*, a complement regulatory molecule that protects against complement‐mediated injury [29]; *Malat1*, a gene shown to regulate inflammation in autoimmune diseases [30]; and *Lncpint*, which is associated with immune homeostasis and inflammation control [31]. Together, these features suggest that syngeneic grafts are associated with a more tissue‐protective immune state. In contrast, genes elevated in allogeneic INs were associated with stronger adaptive immune and pro‐inflammatory responses, like *S1pr5*, linked to T cell trafficking, *Hlf*, linked to promoting pro‐inflammatory activity in CD4+ T Cells, and *Ido2*, which has been implicated as a pro‐inflammatory mediator of B cell responses [32, 33, 34]. Principal component analysis‐based visualization of this gene signature showed clear separation of the two transplant settings across both time points (Figure 1v), demonstrating that IN captures transcriptomic states associated with allogeneic relative to syngeneic groups. The changes in IN‐based immune transcriptome were reflective of the biology within the transplanted islet graft. We mapped the IN‐derived transcriptomic signature onto a published single‐cell RNA sequencing dataset of syngeneic and allogeneic islet grafts collected at day 7 post‐transplant [35]. Analysis of the graft dataset identified diverse immune and stromal populations, including macrophages, dendritic cells, T cell subsets, islet cells, endothelial cells, and mesenchymal cells from both the allogeneic and syngeneic transplant settings (Figure 1w,x). We then generated an IN‐based signature score across graft cell populations by calculating the difference between the mean expression of allogeneic‐enriched and syngeneic‐enriched genes in the IN signature. Strikingly, the IN‐based signature score was significantly elevated across the majority of cell types in the allogeneic relative to syngeneic condition (Figure 1y). This suggested that the transcriptomic state captured by distant subcutaneous IN closely mirrored that within the transplanted islet graft, supporting that IN can capture many aspects of the islet graft‐associated immune dysregulations.

Pathway‐level analysis of the IN by gene set enrichment indicated enrichment of lymphoid and IFN‐γ inflammatory programs in allogeneic condition (Figure 1z and Table S4). In contrast, the syngeneic group was enriched for neutrophil, TGF‐β, TNF‐α/IL‐6, and NOD‐like receptor signaling pathways, suggesting a mixed inflammatory and tissue‐associated immune response. Similar pathway trends were observed at the graft site as well (Figure S3). Together, these data indicate that IN captures not only cellular and gene‐level differences, but also broader immune programs associated with allogeneic rejection versus syngeneic engraftment.

We subsequently evaluated the specificity of the IN signatures for graft rejection by incorporating baseline controls to distinguish IN material‐induced immune responses from rejection‐associated changes. To establish the baseline immune response associated with IN material, we profiled INs explanted on day 14 from healthy C57BL/6 mice and streptozotocin‐induced diabetic C57BL/6 mice without islet transplantation (Figure S4a,b and Table S1). Overall CD45+ immune cell infiltration was comparable across all groups, indicating that transplantation did not simply increase total immune cell recruitment to the IN (Figure S4c and Table S2). Instead, flow cytometric analysis demonstrated remodeling of the IN immune composition following transplantation. Compared with the baseline controls, syngeneic and allogeneic transplantation resulted in changes across both innate and adaptive immune cell populations. Syngeneic recipients exhibited altered neutrophil, macrophage, and dendritic cell frequencies relative to one or both baseline groups, whereas allogeneic recipients had differences primarily within the adaptive immune compartment, including CD4+ T cell and B cell populations (Figure S4d–j). Consistent with these cellular differences, PCA using the gene signature showed distinct transcriptomic shifts in both syngeneic and allogeneic transplant groups relative to the baseline controls, with some overlap among groups (Figure S4k). We further examined whether IN‐liver immune correlations occurred in the absence of transplantation at the day 14 endpoint (Table S3). No significant correlations were detected in healthy C57BL/6 controls, indicating that correlation between immune populations in the IN and liver graft site is not an intrinsic feature of IN implantation (Figure S5). In contrast, STZ‐induced diabetic mice showed significant correlations for some immune populations like neutrophils, macrophages, and B cells, consistent with the ability of the IN to capture immune perturbations related to diabetes onset. Together, these findings demonstrate that the immune signatures identified by the IN primarily reflect allogeneic graft‐associated immune rejection rather than the baseline foreign body response to the PCL IN implantation.

### 2.2 IN Identifies Innate Immune Signatures of Allograft Acceptance vs Rejection Under Anti‐CD40L Therapy

We next investigated immune differences within IN in the context of immunosuppression by comparing accepted and rejected islet allografts under anti‐CD40L immunotherapy. Streptozotocin‐induced diabetic C57BL/6 mice received allogeneic islets (BALB/c to C57BL/6) together with three 500 µg doses of anti‐CD40L on days 0, 7, and 14. INs were implanted subcutaneously at the time of transplantation and serially biopsied with replacement from day 7 until day 70 or onset of hyperglycemia (Figure 2a). Most anti‐CD40L‐treated recipients (14/17) showed reduced blood glucose after transplantation, accepted the graft, and maintained normoglycemia through day 70 (Figure 2b,c and Table S1). A few mice (n = 3) that were observed long‐term showed an increase in blood glucose and graft rejection only after 100 days post‐transplant (Figure 2c). In contrast, anti‐CD40L‐treated rejecting allograft recipients exhibited an initial restoration of normoglycemia followed by recurrent hyperglycemia by day 14 (Figure 2c). We analyzed the IN immune composition from anti‐CD40L accepted and rejected allotransplant groups at day 14 using spectral flow cytometry (Table S2). Relative to the accepted group, INs from the rejected group had a higher proportion of total immune cells, consistent with higher immune recruitment related to rejection (Figure 2d). This increase was accompanied by increased neutrophil proportion, suggesting a more inflammatory niche environment (Figure 2e). In contrast, INs from accepted allografts were enriched for macrophages, particularly CX3CR1+ and CD80+ macrophages, suggesting that graft acceptance is associated with specialized myeloid programs involving immune regulation, tissue surveillance, and antigen‐presenting function (Figure 2f–h). A similar trend was observed for total dendritic cells and CX3CR1+ dendritic cells, which were also higher in INs in the accepted group (Figure 2i,j). No significant differences were observed in total T cells or in proportions of CD4+ and CD8+ T cells between the groups (Figure 2k–m). Accepted allografts also showed higher frequencies of total B cells and CD43+ B cells, indicating additional adaptive immune features associated with durable graft survival (Figure 2n,o). Interestingly, multiple immune populations in the IN significantly correlated with those in the liver graft site at the endpoints (days 70–77) of the accepted anti‐CD40L‐treated group (Figure S6 and Table S3). While total immune‐cell frequencies were not significantly correlated between the IN and liver graft site, neutrophil frequencies exhibited strong positive correlations (Figure S6a,b). Notably, macrophages and CX3CR1+ macrophages, which showed positive correlations in the untreated allogeneic recipients, had inverse correlations following anti‐CD40L treatment, highlighting a treatment‐dependent relationship between immune responses at the IN and graft site (Figure S6c,d). A similar reversal in correlation direction was observed for dendritic cells, especially CX3CR1+ subtypes, although these associations were weaker and did not reach statistical significance (Figure S6e,f). A significant positive correlation was observed for T cell frequencies between the IN and liver graft site, but not for B cells (Figure S6g,h). Together, these findings demonstrated that the IN captures many graft‐associated immune changes following anti‐CD40L treatment.

**FIGURE 2:**
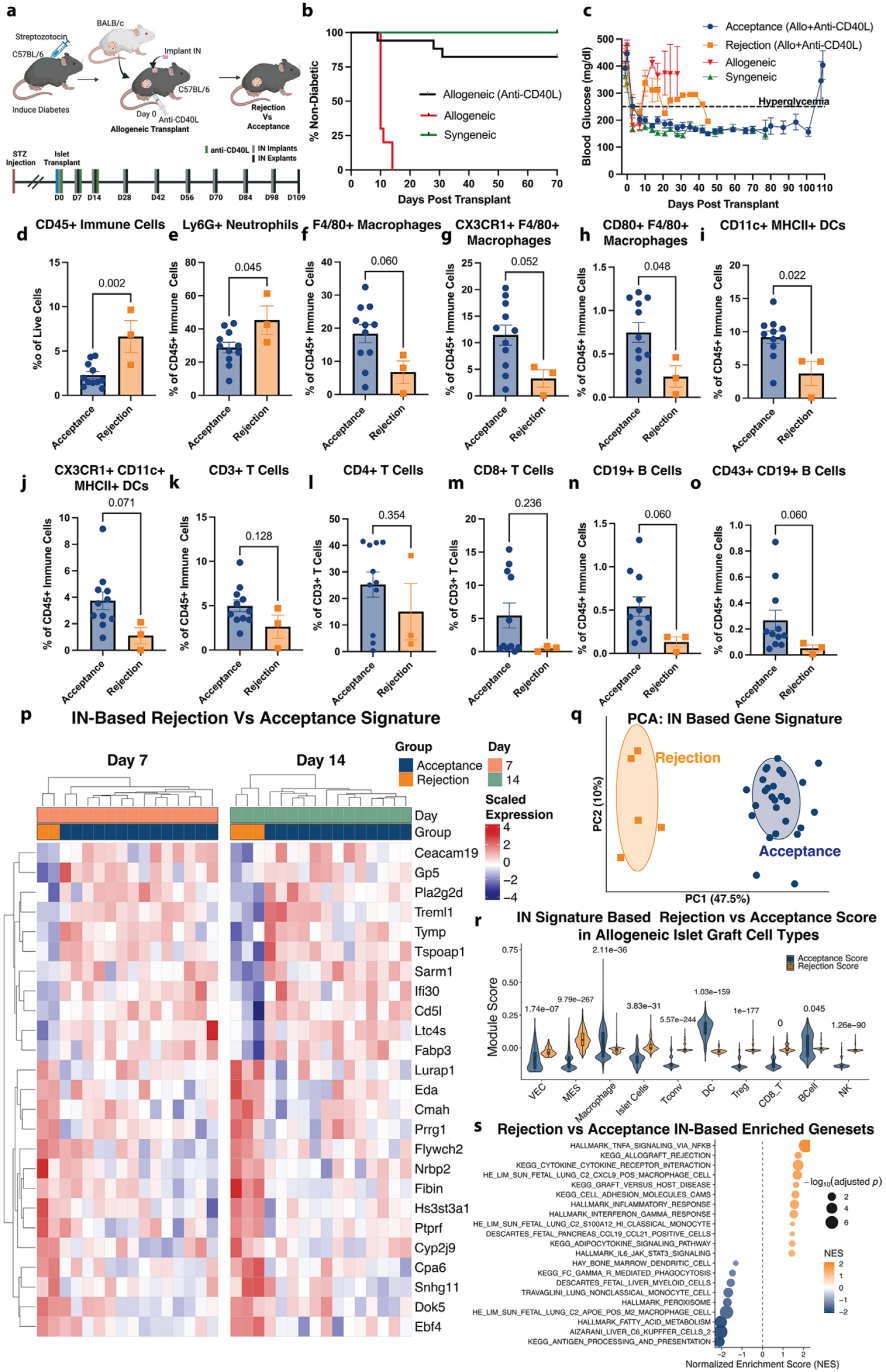
IN identifies innate immune programs distinguishing allograft acceptance from rejection under anti‐CD40L therapy. (a) Experimental design: Streptozotocin‐induced diabetic C57BL/6J mice received allogeneic (BALB/c) islet transplants with concurrent subcutaneous IN implantation and anti‐CD40L treatment. INs were longitudinally biopsied with replacement until day 70 post‐transplant or onset of hyperglycemia. A subset of mice (n = 3) was monitored through day 109 post‐transplant. (b) Diabetes‐free survival in allogeneic (n = 10), syngeneic (n = 7), and allogeneic + anti‐CD40L (n = 17) groups. (c) Blood glucose trajectories of accepted (n = 14) and rejected (n = 3) anti‐CD40L‐treated allografts. Proportions of (d) immune cells, (e) neutrophils, (f) macrophages, (g) CX3CR1+ macrophages, (h) CD80+ macrophages, (i) dendritic cells, (j) CX3CR1+ dendritic cells, (k) T cells, (l) CD4+ T cells, (m) CD8+ T cells, (n) B cells, and (o) CD43+ B cells in accepted (n = 11) and rejected (n = 3) INs at day 14, shown as mean ± SEM. Statistical comparisons used unpaired *t*‐tests or Mann‐Whitney tests based on normality. (p) Heatmap and (q) PCA showing elastic net‐derived transcriptomic signature separating rejection (n = 2–3 per time point) and acceptance (n = 13–14 per time point) samples across days 7 and 14. Ellipses represent 70% confidence intervals. (r) IN‐signature‐based acceptance and rejection scores across allograft cell types. (s) GSEA of IN highlighting selected pathways differentially enriched between accepted and rejected conditions.

We identified that IN‐based immune transcriptomic changes are reflective of biological differences between accepted and rejected allografts under anti‐CD40L immunotherapy. Bulk RNA sequencing was performed on IN biopsies collected at days 7 and 14. Following preprocessing and filtering, elastic net feature selection (α = 0.3) identified a 25‐gene signature that distinguished graft acceptance from rejection at both days 7 and 14, with the direction of most genes conserved across time (Figure 2p and Figure S7). Genes enriched in the accepted group included *Pla2g2d*, an immunoregulatory lipid mediator related to M2‐like macrophages [36]; *Treml1*, associated with myeloid‐related inflammation [37]; *Ltc4s*, involved in leukocyte inflammatory mediator pathways [38]; *Cd5l*, associated with macrophage‐mediated immune regulation [39]; and *Ifi30*, an antigen‐processing gene promoting M2‐like macrophage polarization [40]. Collectively, these genes suggest that graft acceptance is characterized by a controlled myeloid state that integrates immune surveillance, antigen handling, and inflammation regulatory programs. In contrast, rejecting grafts showed higher expression of genes, including *Lurap1*, linked to activation of the canonical NF‐kappa‐B pathway and pro‐inflammatory cytokine production [41], and *Ebf4*, a regulator of cytotoxic functions in immune cells [42]. Principal component analysis‐based visualization of this signature demonstrated clear separation of accepted and rejected samples across both time points (Figure 2q), highlighting the ability of IN to resolve early transcriptomic states linked to divergent allograft outcomes under anti‐CD40L therapy.

The IN‐based acceptance versus rejection signature under anti‐CD40L was also reflected within the islet allograft microenvironment, particularly in myeloid compartments. We mapped the IN‐derived signature onto single‐cell RNA sequencing data from allogeneic islet grafts collected at day 7 post‐transplant [35]. Acceptance and rejection scores were calculated across all allograft cell populations using module scores derived from IN signature genes enriched in each state. Notably, macrophages and dendritic cells exhibited higher acceptance scores than rejection scores (Figure 2r), indicating that myeloid programs identified in IN are recapitulated at the graft site. This finding was consistent with our preceding flow cytometry data showing enrichment of anti‐inflammatory CX3CR1+ macrophages and dendritic cells in accepted grafts. Functional relevance of these myeloid states was further supported by gene set enrichment analysis of the IN in anti‐CD40L‐treated accepted vs rejected allograft groups (Table S4). Rejected grafts showed relatively higher enrichment of inflammatory pathways, including TNF‐α and interferon‐γ signaling, together with signatures of classical monocytes and CXCL9‐associated inflammatory macrophages (Figure 2s). In contrast, accepted grafts were enriched for nonclassical monocytes, M2‐like macrophages, and antigen‐presentation programs, supporting a model in which graft survival is associated with regulated and anti‐inflammatory myeloid immunity.

### 2.3 Allograft Rejection Under Anti‐CD40L is Innate Immune‐Associated Whereas Rejection in Untreated Controls is T Cell‐Associated

We next investigated the distinct immune mechanisms driving allograft rejection with and without anti‐CD40L immunotherapy. INs from anti‐CD40L‐treated rejected allografts were compared with INs from untreated rejected allografts in the C57BL/6 model (Figure 3a). Flow cytometry analysis at day 14 revealed that INs from mice with anti‐CD40L‐treated rejected allografts contained higher frequencies of neutrophils and trends toward increased CD80+ and CCR2+ dendritic cells than untreated controls (Figure 3b–d), indicating a rejection program dominated by innate inflammatory recruitment and activated antigen‐presenting cells. In contrast, INs from the untreated control group had higher proportions of B cells, CD4+ T cells, and CD8+ T cells (Figure 3e–g), consistent with a more lymphocyte‐associated rejection response when anti‐CD40L is absent. Together, these findings suggest that anti‐CD40L reshapes alloimmune rejection by shifting the dominant biology from adaptive T cell responses toward innate immune mechanisms.

**FIGURE 3:**
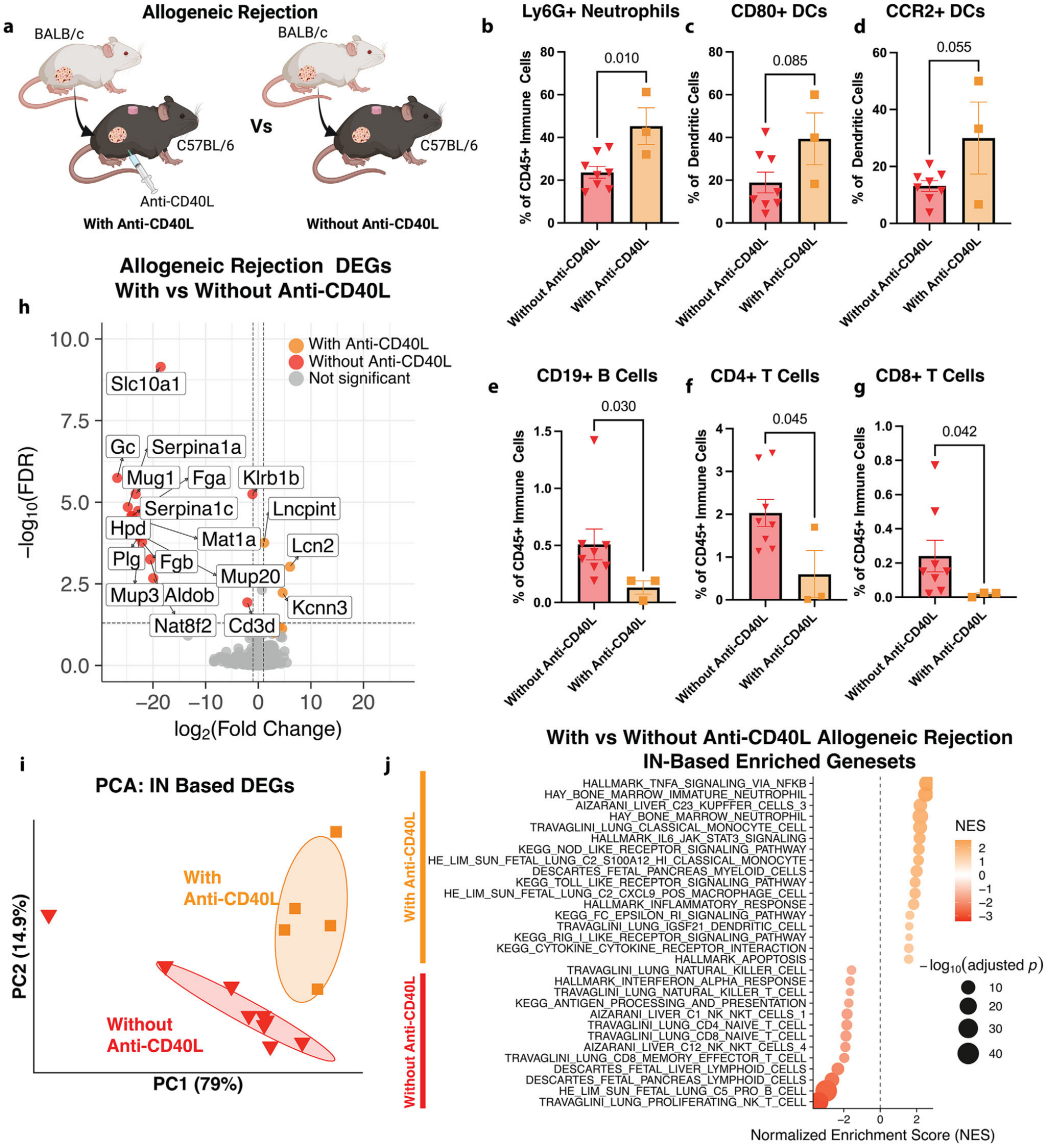
Allograft rejection under anti‐CD40L is innate immune‐associated vs T cell‐associated rejection in control. (a) Experimental design: INs from anti‐CD40L‐treated rejected allografts (days 7 and 14) were compared with untreated allogeneic controls. (b–g) Spectral flow cytometry of INs at day 14 reveals distinct immune composition between anti‐CD40L‐treated (n = 3) and untreated (n = 8) conditions, including (b) neutrophils, (c) CD80+ dendritic cells, (d) CCR2+ dendritic cells, (e) B cells, (f) CD4+ T cells, and (g) CD8+ T cells. Statistical comparisons were performed using unpaired t‐tests (b, c, d, f) or Mann‐Whitney tests (e, g) based on Shapiro‐Wilk normality testing, with data shown as mean ± SEM. (h) Volcano plot highlighting differentially expressed innate and adaptive immune genes between anti‐CD40L‐treated rejected allografts (n = 2–3 per time point) and untreated controls (n = 4–5 per time point) at days 7 and 14 combined. Axes truncated for visualization. (i) PCA of differentially expressed genes showing separation between groups (70% confidence ellipses). (j) Selected differentially enriched pathways between anti‐CD40L‐treated rejection and control allogeneic conditions from gene set enrichment analysis.

Transcriptomic profiling further confirmed that rejection under anti‐CD40L was more innate immune‐associated, whereas rejection in untreated control allografts was dominated by adaptive immunity. Differential gene expression analysis of IN biopsies collected at days 7 and 14 identified genes associated with distinct rejection programs (|log2FC| ≥ 1, padj ≤ 0.1, Figure 3h and Table S5). Untreated control allografts showed higher expression of adaptive immune genes such as *Cd3d* and *Klrb1b*, consistent with T‐ and lymphocyte‐mediated rejection. In contrast, INs from anti‐CD40L‐treated rejected allografts were enriched for innate immune genes, including *Lcn2* and *Lncpint*, supporting the persistence of a myeloid‐related inflammatory state despite costimulatory blockade. PCA using the differentially expressed genes showed distinct clustering of anti‐CD40L‐treated rejected allografts from control untreated allografts (Figure 3i).

Gene set enrichment analysis reinforced the differential rejection programs (Table S4). Anti‐CD40L‐treated rejected allografts showed higher enrichment of neutrophil, classical monocyte, and CXCL9‐associated inflammatory macrophage programs, together with TNF‐α inflammatory pathways (Figure 3j). In contrast, untreated control allografts were enriched for lymphoid programs including CD4+ T cells, CD8+ T cells, B cells, and NKT cell gene sets. Collectively, these results demonstrate that anti‐CD40L alters the dominant biology of rejection from adaptive lymphocyte responses toward innate inflammatory mechanisms, highlighting the ability of IN to resolve mechanistically distinct pathways of graft failure.

### 2.4 Allograft Acceptance Under Anti‐CD40L has a Distinct Immune Signature Over Time From Syngeneic Controls

We investigated differences in immune state of graft acceptance under anti‐CD40L from control syngeneic graft tolerance, to identify residual susceptibility to future rejection. Longitudinal IN biopsies from anti‐CD40L‐treated accepted allografts were therefore compared with INs from syngeneic controls in the C57BL/6 model (Figure 4a). Flow cytometry of INs was performed at days 14, 42, and 70 to define how immune states evolved over time in the two groups. INs from accepted allografts showed a progressive increase in total immune cell abundance over time (Figure 4b), suggesting increasing immune engagement despite preserved graft function. Neutrophils declined over time in the syngeneic group but remained relatively stable in accepted allografts (Figure 4c), consistent with a persistent inflammation profile in the allogeneic acceptance group. Dynamic changes were also observed in other myeloid compartments. In the syngeneic group, the proportion of total macrophages increased over time, including anti‐inflammatory CX3CR1+ macrophages. In contrast, accepted allografts had a decline in total macrophages over time, whereas CX3CR1+ macrophages decreased from days 14 to 42 before returning to earlier levels by day 70 (Figure 4d, e). A similar pattern was observed for dendritic cells. INs from syngeneic controls demonstrated a gradual increase in dendritic cell frequencies over time, including an increase in anti‐inflammatory CX3CR1+ dendritic cells. In contrast, INs from accepted allografts maintained relatively stable frequencies of these populations at lower frequencies (Figure 4f, g). CD4+ T cells increased similarly in both groups, and CD8+ T cells showed a modest increase in accepted allografts compared with syngeneic controls (Figure 4h,i). To further assess the evolution of adaptive immune cell long‐term post anti‐CD40L treatment, we longitudinally monitored INs from anti‐CD40L treated accepted allografts through day 175 in an independent cohort (n = 5; Figure S8). Graft function was maintained throughout this period in these mice, as indicated by blood glucose levels (Figure S8a). While total immune‐cell abundance at the IN showed an increasing trend beginning at day 42, adaptive immune‐cell frequencies showed significant increase primarily at later time‐points, with B cells increasing from day 106 and CD4+ and CD8+ T cells showing significant expansion by day 175 (Figure S8b–e). Together, these findings demonstrate that while anti‐CD40L supports long‐term graft acceptance by initially suppressing the adaptive immune cell expansion, it does not maintain the quiescent immune state that was observed in the syngeneic controls.

**FIGURE 4:**
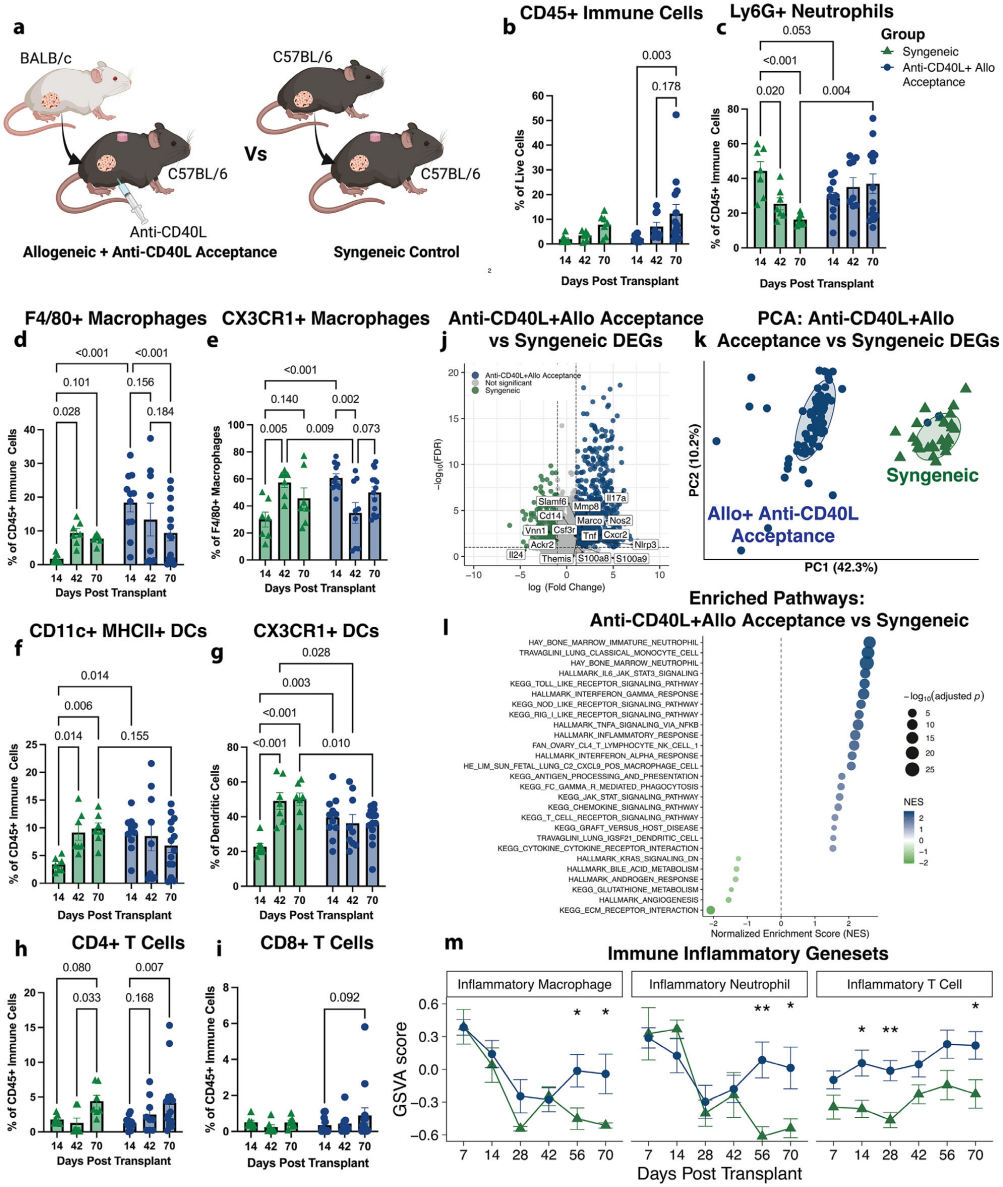
Allograft acceptance under anti‐CD40L has distinct immune signatures from syngeneic controls. (a) Experimental design: INs from anti‐CD40L‐treated accepted allografts (days 7, 14, 28, 42, 56, and 70) were compared with syngeneic controls. (b–i) Longitudinal immune profiling of INs at days 14, 42, and 70 showing temporal immune changes between syngeneic (n = 7 per time‐point) and anti‐CD40L treated accepted allograft (n = 9–14 per time‐point) groups, including (b) immune cells, (c) neutrophils, (d) macrophages, (e) CX3CR1+ macrophages, (f) dendritic cells, (g) CX3CR1+ dendritic cells, (h) CD4+ T cells, and (i) CD8+ T cells. Statistical analysis used mixed‐effects models with Tukey's multiple‐comparison test. Data shown as mean ± SEM. (j) Volcano plot of differentially expressed innate and adaptive immune genes between anti‐CD40L‐treated accepted allografts (n = 11–14 per time‐point) and syngeneic controls (n = 4–5 per time‐point) across days 7–70. Axes truncated for visualization. (k) PCA of differentially expressed genes with 70% confidence ellipses showing separation between groups. (l) Selected enriched pathways from GSEA of IN between anti‐CD40L‐treated accepted and syngeneic conditions. (m) Bulk RNA sequencing‐based sample‐averaged GSVA scores for inflammatory macrophage, neutrophil, and T cell transcriptional gene sets at IN across days 7–70. Welch's t‐test performed at each time point. Mean ± SEM shown.

Transcriptomic differences between INs from anti‐CD40L‐treated accepted allografts versus syngeneic controls further demonstrated that the accepted allograft group remains immunologically distinct from the syngeneic group. Differential gene expression analysis of longitudinal IN biopsies from day 7 to 70 identified differentially expressed genes between the two groups (|log2FC| ≥ 1, padj ≤ 0.1; Figure 4j and Table S6). Accepted allografts had higher expression of both innate and adaptive immune genes, including pro‐inflammatory cytokine signaling genes such as *Il17a*, *Tnf*, and *Nos2*; neutrophil recruitment and activation genes such as *Cxcr2*, *Mmp8*, and *Csf3r*; innate macrophage‐associated genes such as *Marco* and *Cd14*; and a T cell signaling gene, *Themis* [43, 44, 45, 46, 47, 48, 49, 50, 51]. Principal component analysis‐based visualization of these differentially expressed genes demonstrated clear separation between accepted allografts and syngeneic controls (Figure 4k), indicating persistent immune divergence despite maintained graft function.

Pathway‐level analysis of the IN supported immune divergence between anti‐CD40L‐treated accepted allografts versus syngeneic controls (Table S4). INs from accepted allografts were enriched for gene sets related to interferon, IL‐6, and TNF‐α inflammatory signaling, together with antigen presentation, neutrophil, T cell receptor, and classical monocyte programs, indicating a higher immune inflammatory state (Figure 4l). In contrast, syngeneic controls were enriched for angiogenesis, consistent with tissue repair and stable graft integration (Figure 4l). To further examine longitudinal immune trajectories, we quantified gene set variation scores for inflammatory macrophage, neutrophil, and T cell gene sets over time (Table S7). Inflammatory macrophage and neutrophil programs were initially similar between groups but began to diverge at later time points, particularly from day 56 onward, increasing in the accepted allograft group while declining in syngeneic controls (Figure 4m). This pattern suggests that accepted allografts may remain at risk of future rejection rather than progressing toward full immune tolerance. In addition, inflammatory T cell signatures remained consistently higher in accepted allografts throughout the study, further supporting the presence of sustained alloimmune activity despite apparent allograft acceptance (Figure 4m).

### 2.5 IN Captures Immune Biomarkers of Auto‐Allogeneic Transplant Rejection

We next incorporated the autoimmune component of clinical islet transplantation by performing allogeneic islet transplantation in diabetic autoimmune NOD mice (C57BL/6 to NOD), thereby modeling the combined alloimmune and autoimmune response faced by the transplanted grafts (Figure 5a). INs were implanted at a prediabetic stage. In this more stringent setting, treatment with three doses of anti‐CD40L alone was insufficient, and recipients uniformly rejected the graft by day 14 (Figure 5b). In contrast, intensification of immunotherapy with additional rapamycin (2 mg/kg, 15/20 doses) or a fourth anti‐CD40L dose improved graft outcomes and significantly delayed rejection (Figure 5b). Blood glucose trajectories in the anti‐CD40L plus rapamycin groups further revealed two biologically distinct response patterns: an early rejection group that rejected the graft and became hyperglycemic by day 33 and a late rejection group that rejected after day 41 (Figure 5c and Table S1). We profiled IN immune composition at day 14 to detect early immune compositions associated with these divergent outcomes. For both groups, INs were predominantly composed of myeloid cells, particularly neutrophils, macrophages, and dendritic cells, with smaller contributions from T and B cells and no significant differences in overall immune cell frequencies between groups (Figure 5d–i). These findings also suggested that conventional immune composition alone may not fully explain divergent graft trajectories in the auto‐allogeneic setting, motivating deeper transcriptomic interrogation of IN.

**FIGURE 5:**
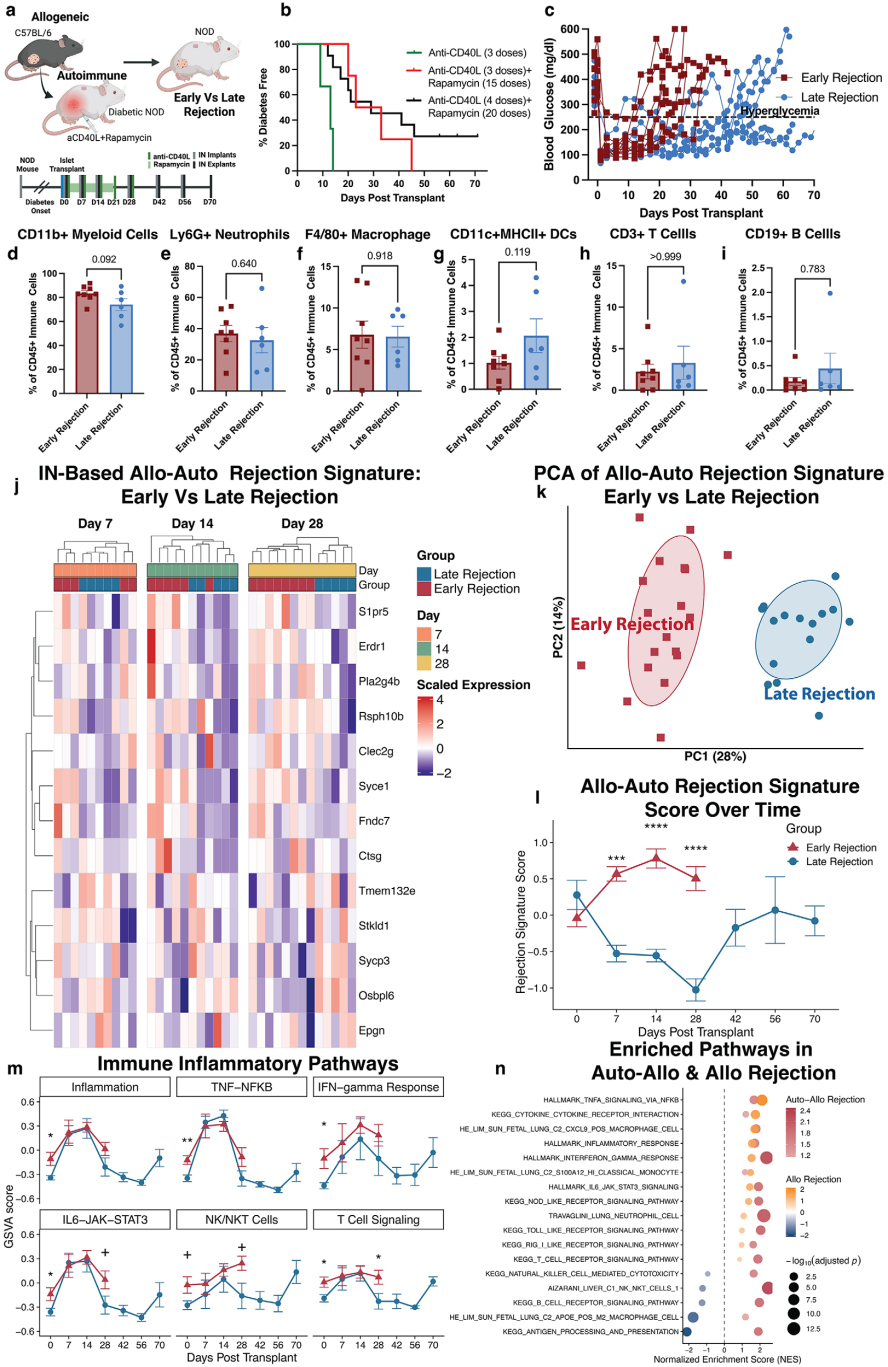
INs capture immune transcriptomic signatures associated with auto‐allogeneic rejection. (a) Experimental design: Prediabetic NOD mice received subcutaneous IN implantation before diabetes onset, followed by allogeneic islet transplantation (C57BL/6J) after diabetes onset with anti‐CD40L and rapamycin treatment. INs were longitudinally biopsied with replacement until day 70 post‐transplant or onset of hyperglycemia. (b) Diabetes‐free survival for anti‐CD40L alone (3 doses, n = 3), anti‐CD40L (3 doses) + rapamycin (15 doses, n = 4), and anti‐CD40L (4 doses) + rapamycin (20 doses, n = 11). (c) Blood glucose trajectories identifying early (≤day 33, n = 9) and late (≥day 41, n = 6) rejection. Proportions of (d) myeloid cells, (e) neutrophils, (f) macrophages, (g) dendritic cells, (h) T cells, and (i) B cells in the IN from early (n = 8) and late (n = 6) rejection groups at day 14. Data are shown as mean ± SEM. Statistical comparisons used unpaired t‐tests or Mann‐Whitney tests based on normality. (j) Heatmap and (k) PCA showing a 13‐gene elastic net signature separating early (n = 5–8 per time‐point) and late (n = 5 per time‐point) rejection across days 7, 14, and 28. Ellipses represent 70% confidence intervals. (l) Longitudinal rejection scores derived from the 13‐gene signature (n = 4–8 per group per time‐point). (m) Bulk RNA sequencing‐based GSVA pathway scores for inflammation‐ and immune‐associated transcriptional gene sets across days 0–70. Welch's t‐test performed at each time point. Mean ± SEM shown. (n) Comparison of selected immune pathways between auto‐allogeneic and allogeneic rejection.

Transcriptomic profiling of IN from early‐ and late‐rejection groups identified a distinct auto‐allo rejection signature associated with divergent graft outcomes. Following preprocessing and filtering of IN bulk RNA sequencing data from days 7, 14, and 28, elastic net feature selection (*α* = 0.6) defined a 13‐gene signature that separated early rejection from late rejection in the autoimmune‐allogeneic setting, with separation becoming progressively more distinct over time (Figure 5j and Figure S9). Genes enriched in early rejection included *S1pr5*, linked to T cell trafficking [32]; *Erdr1*, associated with immune regulation [52]; *Pla2g4b*, involved in inflammatory lipid metabolism and immune cell activity [53]; and *Clec2g*, related to NK cell receptors [54], together suggesting heightened lymphoid immune activation in the early rejection group. Principal component analysis‐based visualization of this signature demonstrated clear separation between the two outcome groups (Figure 5k), indicating that IN captures early molecular programs linked to the timing of graft failure. When applied across transplant settings, the 13‐gene signature largely separated untreated allogeneic from syngeneic recipients by PCA but did not distinguish accepted from rejected allografts under anti‐CD40L treatment (Figure S10). These findings indicate that the signature is context‐dependent and more reflective of rejection associated with stronger adaptive immune responses rather than the more innate‐associated rejection observed in the anti‐CD40L‐treated allogeneic model.

We generated an early‐rejection score using gene set variation analysis based on the relative enrichment of early rejection‐associated IN signature genes to track rejection dynamics over time (Table S8). In the early‐rejection group, the score progressively increased and was already elevated by day 28, indicating imminent graft loss. In contrast, the late‐rejection group had an initial decline in the score through day 28, followed by a subsequent rise prior to graft failure (Figure 5l). Temporal divergence and later re‐emergence of this score highlight the ability of IN not only to distinguish rejection phenotypes, but also to longitudinally detect evolving immune risk before overt loss of graft function. We next examined pathway‐level dynamics to investigate the biology underlying evolution of immune programs in early‐ and late‐rejection groups. Notably, the GSVA scores for inflammation, TNF‐NF‐κB, interferon, IL‐6‐JAK‐STAT, NK/NKT cell, and T cell signaling gene sets were higher at baseline in the early‐rejection group than in the late‐rejection group, suggesting a more primed immune state before transplantation (Figure 5m). Following transplantation and anti‐CD40L and rapamycin treatment, these pathways became comparable between groups at days 7 and 14, consistent with an initial therapeutic suppression of immune activation. However, by day 28, several pathways, including IL‐6‐JAK‐STAT, NK/NKT, and T cell signaling, showed an increase in the early‐rejection group, whereas the rise in the late‐rejection group was delayed until closer to day 70. These temporal differences indicate that the early rejection group has faster‐increasing inflammatory and cytotoxic programs as compared to the late rejection group.

We next investigated the potential for IN to resolve immune components associated with alloimmune versus autoimmune responses to graft rejection under immunotherapy. Pathway enrichment profiles derived from rejection‐associated IN genes in anti‐CD40L‐treated allografts (rejection versus acceptance) were compared with those from anti‐CD40L plus rapamycin‐treated auto‐allografts (early versus late rejection) (Figure 5n and Table S4). Interestingly, pathways related to TNF‐α inflammation, inflammatory response, interferon signaling, CXCL9‐associated macrophages, IL‐6, neutrophils, and classical monocytes were similarly enriched in both allo‐only and auto‐allo settings, suggesting that these myeloid inflammatory programs are core features of alloimmune graft injury under immunotherapy. In contrast, pathways related to T cells, NK‐cell cytotoxicity, B cells, and antigen presentation were preferentially enriched in the auto‐allogeneic setting, indicating that the added lymphoid component may reflect the autoimmune component of rejection. Together, these findings demonstrate that IN can deconvolute overlapping rejection biology and distinguish innate inflammatory programs primarily associated with alloimmunity from adaptive immune programs linked to autoimmunity.

## 3 Discussion

Monitoring of islet transplantation remains one of the most challenging areas in transplantation medicine. Unlike vascularized solid organs like heart or kidney that are transplanted as a discrete anatomical unit, islets are infused as dispersed cellular clusters, most commonly into hepatic portal circulation, where they engraft as microscopic deposits distributed throughout the liver [55]. This diffuse and inaccessible architecture makes direct imaging and tissue biopsy highly challenging, preventing routine histological assessment of graft health [17, 56]. Current clinical measures, including blood glucose, C‐peptide, HbA1c, and glucose tolerance testing, act largely as functional readouts and often reflect damage after considerable loss of viable graft mass has already occurred [57]. While approaches such as blood gene expression profiling and plasma donor‐derived cell‐free DNA have shown promise in other transplant settings, their application to islet transplantation remains constrained by low tissue mass, dispersed engraftment, and uncertain sensitivity for early injury [58, 59]. Here, we report an engineered immunological niche that serves as a minimally invasive cell‐capture platform for longitudinal monitoring of transplant‐associated immune responses in two clinically relevant models: allogeneic transplantation in C57BL/6 mice under anti‐CD40L, and auto‐alloimmune transplantation in NOD mice under anti‐CD40L plus rapamycin.

The IN has strong potential to capture major immune dysregulations associated with the transplanted islet grafts. The transcriptomic signature derived from IN that distinguished allogeneic from syngeneic transplantation was reflective of immune dysregulation. Several genes within this signature highlighted distinct graft environments. *Cd59b*, elevated in syngeneic transplantation, encodes a complement regulatory molecule that may protect islets from complement‐mediated injury [60]. *Malat1* and *Lncpint*, both linked to inflammatory responses, were also higher in syngeneic grafts, suggesting that even syngeneic transplantation induces an early inflammatory signaling [61]. However, longitudinal analysis showed that this inflammation resolved over time in the syngeneic group, suggesting tolerance. In contrast, the allogeneic group displayed a more adaptive immune‐associated rejection profile. *S1pr5*, involved in lymphocyte trafficking, *Hlf*, associated with pro‐inflammatory CD4+ T cell activity, and *Ido2*, linked to pro‐inflammatory B cell responses, were enriched in allogeneic transplantation [32, 33, 34]. Overall, IN gene signature‐based scores were higher across most immune cell populations in allogeneic grafts than in syngeneic grafts, spanning both innate and adaptive compartments. Importantly, no significant immune cell correlations were observed between the IN and liver graft site in healthy mice without transplantation, whereas significant correlations emerged in the transplant recipients, especially in the allogeneic group. This context‐dependent correlation indicates that IN mirrors immune and related transcriptomic differences between allografts and syngeneic grafts and is indicative of graft‐associated immune biology, rather than merely reflecting the baseline foreign body response due to the implanted biomaterial. Interestingly, the IN also captured graft‐associated immune cell and transcriptomic changes between graft acceptance and rejection groups in anti‐CD40L‐treated allografts. IN identified macrophages and dendritic cells, particularly tissue‐resident anti‐inflammatory CX3CR1+ subsets, as key immune populations related to allograft acceptance under anti‐CD40L. These cells have established roles in pancreas islet homeostasis and transplant immunity, supporting the biological relevance of the IN findings [62, 63]. The acceptance‐associated IN gene signature included *Pla2g2d*, *Cd5l*, and *Ifi30*, which are linked to immunoregulation, antigen handling, and M2‐like macrophage polarization [36, 39, 40]. Consistent with the IN signature, macrophages and dendritic cells were the only allograft cell types with significantly higher acceptance than rejection scores based on the IN‐derived signature. While the number of anti‐CD40L‐treated grafts that underwent rejection was limited due to the high efficacy of the anti‐CD40L therapy, these findings provide a basis for future validation in larger cohorts. IN also captured adaptive immune programs associated with autoimmune‐mediated rejection in the auto‐allograft setting, consistent with prior reports [64]. Overall, these findings across multiple transplant settings support that IN captures tissue‐associated immune changes reflective of the transplanted islet graft. By capturing rejection‐associated adaptive immune responses in untreated allografts, acceptance‐associated regulatory myeloid programs under anti‐CD40L therapy, and autoimmune rejection programs in the auto‐allograft setting, the IN functions as a minimally invasive platform for longitudinal monitoring of graft immune status and for identifying mechanisms underlying transplant outcomes.

IN provides comprehensive insights into immune programs that distinguish graft outcomes across transplantation settings, resolving signatures of rejection, tolerance, and therapeutic response under anti‐CD40L immunotherapy. IN revealed a shift in the immune landscape under anti‐CD40L treatment, where rejection is less lymphoid‐associated and more characterized by enrichment of innate immune populations, including neutrophils, macrophages, dendritic cells, and their associated subsets. This result is consistent with inhibition of T cell activation by anti‐CD40L costimulatory blockade and aligns with prior studies highlighting the role of innate inflammation in islet transplantation [65, 66, 67]. Macrophages and neutrophils have been shown to mediate allograft rejection through mechanisms such as phagocytosis and trogocytosis, while graft‐infiltrating dendritic cells have promoted rejection by locally presenting donor alloantigens to effector T cells within the graft. Interestingly, in the anti‐CD40L‐treated allograft acceptance group, IN identified subsets of these innate immune cells associated with regulatory and tolerogenic functions, suggesting a more nuanced role in graft outcomes depending on cellular subtypes. Previous reports have also highlighted the importance of regulatory dendritic cells and macrophages in promoting transplant tolerance under co‐stimulatory blockade through immunoregulatory and T cell‐suppressive mechanisms [68, 69]. Together, these findings suggest that IN‐derived immune signatures can resolve distinct innate immune subsets that differentially contribute to rejection versus tolerance. Importantly, IN also captures dynamic immune changes over time within the acceptance group under anti‐CD40L. Despite initial graft acceptance, a progressive increase in inflammatory innate immune signatures was observed, including neutrophil and macrophage‐associated inflammation, relative to syngeneic controls. Notably, this increase is more evident by day 56 post‐transplant in IN, whereas blood glucose levels do not rise until approximately day 100 in animals that eventually reject the graft. This time lag highlights the ability of IN to detect early, subclinical inflammatory changes that precede overt graft failure. Clinically, serial IN sampling during immunosuppression withdrawal could detect re‐emerging alloimmune activity in recipients before graft dysfunction becomes apparent. Such changes could indicate that immunosuppression should be maintained or increased in those patients, whereas a quiescent immune state could support withdrawal of immunosuppression. Overall, IN distinguishes graft acceptance versus rejection while capturing anti‐CD40L‐associated shifts in pro‐inflammatory and regulatory innate immune programs, enabling prediction of transplant outcomes.

In conclusion, we demonstrate that the synthetic IN acts as a minimally invasive cell capture platform, monitoring immune processes that determine islet graft fate in clinically relevant murine models. By identifying early rejection signatures, distinguishing underlying immune mechanisms, and capturing heterogeneity in autoimmune and allograft response to anti‐CD40L immunotherapy, IN addresses major unmet diagnostic challenges in post‐transplant care. Such an approach could provide a window for intervention before irreversible graft loss and guide selection or adjustment of immunotherapies, while reducing reliance on highly invasive graft biopsies and delayed metabolic markers. A limitation of this study is that the immune signatures and pathway analyses rely primarily on transcriptome‐level data. Further multimodal modeling that integrates complementary proteomic analysis of IN could validate these immune signatures and identify additional monitoring targets not captured by transcriptomic analysis alone. The next step toward clinical translation will be to validate these immune signatures in human islet transplantation. These validation studies need to consider differences in foreign body responses across species, which may influence the composition of the IN immune microenvironment. Establishing biomaterial IN safety, implantation feasibility, and clinical biomarker performance will be critical for translating the IN into a clinically useful islet graft‐monitoring platform. Overall, these findings position IN as a promising diagnostic platform for immune monitoring in islet transplantation and provide a foundation for future clinical translation.

## 4 Materials and Methods

### 4.1 Animal Cohort Design

All animal studies were conducted in accordance with institutional guidelines and protocols approved by the University of Missouri Institutional Animal Care and Use Committee. For allogeneic islet transplantation, BALB/c mice as donors and C57BL/6 mice as recipients were used with or without anti‐mouse CD40 ligand (CD40L) monoclonal antibody treatment. Syngeneic transplants utilized C57BL/6 mice for both donors and recipients. For auto‐allogeneic studies, female non‐obese diabetic (NOD/ShiLtJ) mice served as recipients of C57BL/6 donor islets, treated with a combination of anti‐CD40L mAb and rapamycin. For baseline inflammation studies, C57BL/6 mice were rendered diabetic with streptozotocin (STZ; 170 mg/kg) and received an insulin pellet at the time of IN implantation (day 0) to maintain glycemic control through day 14. A separate cohort of unperturbed C57BL/6 mice served as the healthy baseline control. Individual mouse details are provided in Table S1.

### 4.2 Immunological Niche Fabrication

Microporous poly(ε‐caprolactone) (PCL)‐based immunological niches were fabricated by the salt leaching technique as previously described [70]. First, 99 g of NaCl (250 to 425 µm) was mixed with 1 g of PCL pellets for 1 h at 85 °C. Subsequently, around 77.5 mg of PCL‐NaCl melt dispersion was pressed into 5 mm diameter scaffolds at 1500 psi for 45 s. They were then heated at 65°C for 5 min on each side to anneal PCL. The scaffolds were placed in a deionized water bath for salt leaching, and resulting porous niches were sanitized with 70% ethanol immersion followed by sterile PBS rinses.

### 4.3 Immunological Niche Implantation and Retrieval

Mice were anesthetized with 2% isoflurane and maintained under anesthesia via a nose cone throughout all surgical procedures. INs were implanted into the subcutaneous space through a small dorsal incision. To minimize post‐operative pain, mice received carprofen (5 mg/kg, subcutaneous) at the time of surgery and again 24 h post‐operatively and were monitored during recovery for signs of distress. For IN retrieval, mice were re‐anesthetized with 2% isoflurane, and the implant site was shaved and disinfected. A small incision adjacent to the implant was reopened using sterile forceps, and IN was carefully excised and removed. The incision was closed with wound clips. Explanted INs designated for transcriptomics analysis were immediately flash‐frozen in chilled isopentane, maintained on dry ice, and stored at −80 °C until further processing. INs intended for flow cytometry were immediately placed in MACS buffer (500 mL of 1X PBS, 8.3 mL of 30% BSA, 2 mL of 0.5 M EDTA) and stored on ice prior to processing. All experimental groups underwent identical IN implantation and biopsy procedures. Consequently, all longitudinal comparisons were performed relative to matched control groups subjected to the same IN sampling timeline to control for procedure‐associated inflammatory responses.

### 4.4 Flow Cytometry

Explanted INs were processed into single‐cell suspensions for flow cytometric analysis by mechanical dissociation followed by enzymatic digestion. Briefly, INs were minced into small fragments and digested in Liberase TL (Millipore Sigma, Cat. No. 5401020001; 10 U/mL) for 30 min at 37°C. The digested material was passed through a 70‐µm cell strainer, and red blood cells were lysed using ACK buffer (Fisher Scientific, Cat. No. A1049201) for 1 min. Cells were subsequently washed and resuspended in FACS buffer consisting of PBS supplemented with 0.5% bovine serum albumin and 2 mM EDTA. For staining, cells were aliquoted at approximately 1 × 10^6^ cells per tube. Samples were incubated with anti‐CD16/32 Fc receptor blocking antibody (BD Biosciences, Cat. No. 553142; 1:50) for 15 min at 4°C in the dark, followed by surface staining for 30 min at 4°C in the dark with a multicolor antibody panel. Core markers were maintained across experiments, with fluorophore substitutions or panel refinements introduced as needed to optimize spectral resolution between runs. The panel included LIVE/DEAD Fixable Blue Dead Cell Stain (Thermo Fisher Scientific, Cat. No. L23105; 1:400), anti‐mouse CD45 APC‐Fire 810 (clone 30‐F11, BioLegend, Cat. No. 103174; 1:150), anti‐mouse CD3 BUV737 (clone 17A2, Thermo Fisher Scientific, Cat. No. 367‐0032‐82; 1:20) or CD3 V500 (clone 500A2, BD Horizon, Cat. No. 560771; 1:50) or CD3 APC (clone 145‐2C11, BD Pharmingen, Cat. No. 553066, 1:50), anti‐mouse CD4 Alexa Fluor 700 (clone RM4‐5, Thermo Fisher Scientific, Cat. No. 56‐0042‐82; 1:150), anti‐mouse CD19 PE‐eFluor 610 (clone 1D3/CD19, Thermo Fisher Scientific, Cat. No. 61‐0193‐82; 1:100), anti‐mouse F4/80 Pacific Blue (clone BM8, BioLegend, Cat. No. 123124; 1:100), anti‐mouse CD11b BV510 (clone M1/70, BioLegend, Cat. No. 101263; 1:50), anti‐mouse CD11c Alexa Fluor 647 (clone N418, BioLegend, Cat. No. 117312; 1:100), anti‐mouse Ly6G BUV395 (clone 1A8, BD Biosciences, Cat. No. 563978; 1:100), anti‐mouse CD80 APC‐Fire 750 (clone 16‐10A1, BioLegend, Cat. No. 104740; 1:50), anti‐mouse I‐A/I‐E (MHC‐II) Spark Blue 550 (clone M5/114.15.2, BioLegend, Cat. No. 107662; 1:50), anti‐mouse CD43 FITC (clone S11, BioLegend, Cat. No. 143204; 1:20), anti‐mouse Ly6C BV605 (clone HK1.4, BioLegend, Cat. No. 128036; 1:20), anti‐mouse CX3CR1 PE (clone Z8‐50, BD Biosciences, Cat. No. 567530; 1:50), anti‐mouse CCR2 BV421 (clone SA203G11, BioLegend, Cat. No. 150605; 1:100), and anti‐mouse CD86 PE‐Cy7 (clone GL1, Thermo Fisher Scientific, Cat. No. 25‐0862‐82; 1:200). Following staining, cells were washed twice with PBS, resuspended in 300 µL PBS, and acquired on a Cytek Northern Lights Aurora flow cytometer. For each sample, 20,000 or more CD45+ events were collected. Flow cytometry data were analyzed using FlowJo v10.10.0. Single‐stained and fluorescence‐minus‐one (FMO) controls were included for compensation and gating. Samples collected at intermediate days were assigned to the nearest time‐point for analysis. For the long‐term anti‐CD40L cohort (day 175), two day‐70 samples were excluded from flow cytometry analyses due to technical quality‐control failures of discontinuous event acquisition and insufficient total events.

### 4.5 Bulk RNA Sequencing Analysis

Frozen INs were homogenized in 800 µL TRIzol reagent (Fisher Scientific, Cat. No. 15596026) using mechanical disruption at 15,000 rpm. Total RNA was subsequently purified from the lysate using the Direct‐zol RNA Miniprep Plus kit (Fisher Scientific, Cat. No. NC1047980) according to the manufacturer's protocol. RNA quantity and purity were assessed prior to submission to the Advanced Genomics Core at the University of Michigan for bulk RNA sequencing. Sequencing libraries were prepared using poly‐A enrichment workflows for most samples, with a subset processed using ribosomal RNA depletion‐based library preparation. Libraries were sequenced on an Illumina NovaSeq X Plus platform. Raw base call files were converted to demultiplexed FASTQ files using BCL Convert software v4.0 (Illumina).

Bulk RNA‐sequencing count matrices were preprocessed using a custom filtering pipeline for quality control and removal of low‐confidence features, followed by exclusion of undefined transcripts and pseudogenes prior to downstream analysis. Samples collected at intermediate days were assigned to the nearest time‐point for analysis. Terminal samples collected after day 56 were grouped with the day 70 end‐time‐point bin. Differential gene expression analysis was performed using DESeq2 with adjustment for batch, time‐point, and treatment effects wherever applicable [71]. For gene signature derivation, variance‐stabilized gene expression values were batch‐corrected while preserving group‐level differences and adjusting for time‐point as a covariate. Candidate features were restricted to genes meeting differential expression thresholds (p ≤ 0.05 and |log_2_ FC| ≥ 0.5), reducing dimensionality prior to model fitting. An elastic net model was used to classify allogeneic versus syngeneic samples. The parameter α, which controls the balance between L1 and L2 penalties in elastic net, was systematically evaluated across a predefined grid. For each α, regularization strength (λ) was optimized using leave‐one‐out cross‐validation, with model performance assessed by cross‐validated deviance. The optimal parameter α was selected based on minimizing deviance while maintaining enough retained features to balance predictive performance and model interpretability. To ensure robustness of feature selection, stability selection was performed over 1,000 iterations using repeated subsampling of approximately 80% of samples from each class to preserve class balance. Gene selection frequency was computed across iterations, and genes identified in ≥70% of runs were defined as stable features. For allogeneic versus syngeneic comparison, sex chromosome‐associated genes were excluded from the final signature. Gene set enrichment analysis (GSEA) was performed using clusterProfiler, with gene sets obtained from MSigDB, including C8, Hallmark, and KEGG gene set collections [72]. All genes were ranked by the DESeq2 Wald statistic and pathways with adjusted p ≤ 0.1 and absolute normalized enrichment score (|NES|) ≥ 1 were considered significantly enriched. Gene set variation analysis (GSVA) was performed on variance‐stabilized bulk RNA‐seq data following batch correction using limma [73, 74]. For allogeneic acceptance vs syngeneic, GSVA scores were computed using a Gaussian kernel for curated and custom immune gene sets, including inflammatory macrophage, neutrophil, and T cell signatures. For the auto‐allogeneic signature score, GSVA was applied separately to predefined upregulated and downregulated gene sets, and the composite score was calculated as the difference between enrichment scores of upregulated and downregulated genes for each sample. Pathway enrichment scores were calculated per sample and compared across groups and time points using Welch's *t*‐test.

### 4.6 Single Cell RNA Sequencing Analysis

Islet single‐cell RNA‐sequencing data from Chen et al. were reanalyzed using Seurat 5.4.0, with quality control filtering, SCTransform normalization, and PCA/UMAP‐based dimensionality reduction [75]. Clusters were identified using shared nearest neighbor graph‐based clustering and annotated using previously defined canonical marker genes. To quantify IN‐derived immune signatures differentiating allogeneic vs syngeneic in SCT‐normalized single‐cell data, an elastic net‐derived gene signature was applied by computing a per‐cell score as the difference between the average expression of upregulated and downregulated genes in the IN signature. For the allogeneic graft single‐cell data, rejection‐ and acceptance‐associated elastic net gene signatures were scored separately using Seurat's AddModuleScore, and module scores were compared across cell types. Single‐sample gene set enrichment analysis (ssGSEA) was performed using curated MSigDB C8, Hallmark, and KEGG gene sets to estimate pathway activity at the single‐cell level. Differences between conditions were quantified as mean pathway score differences and assessed using Wilcoxon rank‐sum tests with Benjamini‐Hochberg correction.

### 4.7 Statistics

Plots for flow cytometry, blood glucose, and survival were generated in GraphPad Prism v10.6.1 and RStudio, which were also used for statistical analyses. Diabetes‐free survival was evaluated using Kaplan‐Meier analysis. For single time‐point comparisons between two groups, normality was assessed using the Shapiro‐Wilk test; unpaired t‐tests were applied for normally distributed data and Mann‐Whitney tests otherwise. Longitudinal two‐group flow data were analyzed using mixed‐effects models with Tukey's multiple‐comparison test (pooled variance). For multiple group (>2) comparisons, statistics were analyzed using ordinary one‐way ANOVA with Tukey's multiple‐comparisons test and a single pooled variance for normally distributed data, or Kruskal–Wallis test with Dunn's multiple‐comparisons test for non‐normally distributed data. Longitudinal flow cytometry data from long‐term anti‐CD40L‐treated allogeneic recipients (day 175) were analyzed using linear mixed‐effects models, with time as a categorical fixed effect, and the overall effect of time was assessed using an F‐test. Associations between immune population frequencies at the IN and liver graft site from the same animals were assessed using Pearson correlation analysis, performed separately for syngeneic, allogeneic, and allogeneic plus anti‐CD40L recipients.

Bulk RNA‐seq data from IN were analyzed using DESeq2. Log_2_ fold changes were estimated using the Wald test. Genes with |log_2_FC| ≥ 0.5 and p ≤ 0.05 were selected for downstream elastic net feature selection, while differential expression was defined using |log_2_FC| ≥ 1 and adjusted p ≤ 0.1. Gene set enrichment analysis (GSEA) considered pathways significant at adjusted p ≤ 0.1 and |NES| ≥ 1. For GSVA, pathway scores were computed per sample and compared across groups using Welch's t‐test.

For single‐cell RNA‐seq analyses, differences in IN‐signature scores (allogeneic vs syngeneic) and rejection vs acceptance scores within cell types were assessed using Wilcoxon rank‐sum tests with Benjamini‐Hochberg correction. ssGSEA pathway differences were quantified as mean score differences and tested using Wilcoxon rank‐sum tests with Benjamini–Hochberg correction.

## Author Contributions

Conceptualization: JR, AJN, LDS, ESY. Methodology: JR, AJN, MT, AT, SW, BH, YJ, LDS, ESY. Investigation: JR, AJN, MT, AT, BH, LDS, ESY. Visualization: JR, AJN, LDS, ESY. Funding Acquisition: LDS, ESY. Project Administration: LDS, ESY. Supervision: LDS, ESY. Writing – original Draft: JR, LDS, ESY. Writing – review & Editing: JR, AJN, at, BH, LDS, ESY.

## Funding

This work was supported by Breakthrough T1D (formerly JDRF) grants 2‐SRA‐2023‐1454‐S‐B (L.D.S.) and 2‐SRA‐2024‐1480‐S‐B (L.D.S.). This work also received support from NIH grant U01EB036955.

## Conflicts of Interest

The authors declare no conflicts of interest.

## Supporting information




**Supporting File 1**: advs78154‐sup‐0001‐SuppMat.docx.

**Supporting File 2**: advs78154‐sup‐0002‐SuppMat.zip.

## Data Availability

IN bulk sequencing data are available at Gene Expression Omnibus, accession number GSE330749. Islet graft single cell RNA sequencing data can be found at Gene Expression Omnibus, accession number GSE198865. Code used in this study is publicly available on GitHub: https://github.com/shea‐lab/IsletTransplantRejection. The flow cytometry raw data are available in the Deep Blue Repositories, the University of Michigan Library's data repository: https://doi.org/10.7302/bk9v‐a018.
