# Supplementary material for: Immune Biomarkers of Islet Transplant Rejection Revealed by Synthetic Immunological Niche
Source: Adv Sci (Weinh). 2026 Oct 8:e78154. Online ahead of print. doi: 10.1002/advs.78154 (PMC13650018; doi:10.1002/advs.78154)
Supplement: Supplementary file 1 [file ADVS-9999-e78154-s001.docx]

**List of Supplementary Materials**

**Supplementary Figures**

**Sup. Fig. 1: Representative Flow Gating Scheme:**  Gating strategy used for spectral flow cytometry analysis for the immunological niches from C57BL/6 transplants. Gates were slightly adjusted for NOD INs and liver tissues

**Sup. Fig. 2: Gene-level temporal expression profiles of the IN-based Allogeneic vs Syngeneic Signature.** Expression of individual allogeneic vs syngeneic signature genes in IN at Days 7 and 14 following syngeneic and allogeneic islet transplantation. Expression values represent batch-adjusted variance-stabilized (VST) RNA-seq expression. Points represent individual biological replicates, while lines and error bars indicate the group mean ± SEM.

**Sup. Fig. 3: ssGSEA analysis of allogeneic vs syngeneic islet grafts.** Heatmap of differences in mean ssGSEA scores of selected IN-based genesets across cell types in allogeneic vs syngeneic islet grafts. Statistical significance was assessed using a Wilcoxon rank-sum test with Benjamini–Hochberg correction.

**Sup. Fig. 4 Characterization of IN immune response under baseline and transplant conditions.** **(a)** Schematic of experimental groups used to establish baseline IN immune composition, including healthy C57BL/6 mice and STZ-induced diabetic C57BL/6 mice receiving an insulin pellet at the time of IN implantation. **(b)** Blood glucose levels across experimental groups (n=4-5 per group). **(c-j)** Flow cytometric analysis of IN immune composition, including **(c)** CD45+ immune cells, **(d)** Ly6G+ neutrophils, **(e)** F4/80+ macrophages, **(f)** CD11c+MHC-II+ dendritic cells, **(g)** CD3+ T cells, **(h)** CD4+ T cells, **(i)** CD8+ T cells, and **(j)** CD19+ B cells. Points represent individual biological replicates, while lines and error bars indicate the group mean ± SEM. Statistics were analyzed using ordinary one-way ANOVA with Tukey’s multiple-comparisons test and a single pooled variance for normally distributed data, or Kruskal–Wallis test with Dunn’s multiple-comparisons test for non-normally distributed data. **(k)** PCA showing IN allo vs syn transcriptomic signature for day 14. Ellipses represent 70% confidence interval.

**Sup. Fig. 5: IN captures key anti-CD40L associated immune changes in the liver graft site.** Paired Pearson correlation analysis of immune population frequencies between the IN and liver graft site from the same animals at day 14 endpoint, calculated separately for C57BL/6 (n=5) and C57BL/6+STZ (n=4) for **(a)** CD45+ immune cells, **(b)** Ly6G+ neutrophils, **(c)** F4/80+ macrophages, **(d)** CD11c+MHC-II+ dendritic cells, **(e)** CD3+ T cells, and **(f)** CD19+ B cells. Shaded regions represent 95% confidence intervals around the linear regression lines. Pearson’s correlation coefficient (r) and two-sided P values are shown

**Sup. Fig. 6: IN captures key anti-CD40L associated immune changes in the liver graft site.** Paired Pearson correlation analysis of immune population frequencies between the IN and liver graft site from the same animals at study endpoints, calculated separately for allogeneic (n=10) and allogeneic +anti-CD40L (n=10) recipients for **(a)** CD45+ immune cells, **(b)** Ly6G+ neutrophils, **(c)** F4/80+ macrophages, **(d)** CX3CR1+ F4/80+ macrophages, **(e)** CD11c+MHC-II+ dendritic cells, **(f)** CX3CR1+ dendritic cells, **(g)** CD3+ T cells, and **(h)** CD19+ B cells. Shaded regions represent 95% confidence intervals around the linear regression lines. Pearson’s correlation coefficient (r) and two-sided P values are shown

**Sup. Fig. 7: Gene-level temporal expression profiles of the IN-based anti-CD40L treated Allogeneic Acceptance vs Rejection Signature.** Expression of individual anti-CD40l treated allogeneic acceptance vs rejection signature genes in IN at Days 7 and 14 following islet transplantation. Expression values represent batch-adjusted variance-stabilized (VST) RNA-seq expression. Points represent individual biological replicates, while lines and error bars indicate the group mean ± SEM.

**Sup. Fig. 8: Long-term monitoring of immune responses following anti-CD40L-mediated allograft acceptance. (a)** Blood glucose levels in anti-CD40L-treated allogeneic recipients (n = 5) monitored through day 175 post-transplant**. (b-e)** Longitudinal flow cytometric analysis of INs collected at days 14, 28, 42, 70, 106, and 175, showing **(b)** CD45+ immune cells, **(c)** CD4+ T cells, **(d)** CD8+ T cells, and **(e)** CD19+ B cells. Statistics were analyzed using linear mixed-effects models, with time as a categorical fixed effect and the overall effect of time was assessed using an F-test.

**Sup. Fig. 9: Gene-level temporal expression profiles of the IN-based anti-CD40L and rapamycin treated Auto-Allogeneic Early vs Late Rejection Signature.** Expression of individual anti-CD40L and rapamycin treated auto-allogeneic early vs late rejection signature genes in IN at Days 7, 14 and 28 following islet transplantation. Expression values represent variance-stabilized (VST) RNA-seq expression. Points represent individual biological replicates, while lines and error bars indicate the group mean ± SEM.

**Sup. Fig. 10:** **Cross-model conservation of the NOD-derived 13-gene auto-allo rejection signature.** The 13-gene IN signature, derived from early- versus late-rejecting NOD auto-allograft recipients, was evaluated in independent C57BL/6 transplant models. Principal component analysis showed that the signature **(a)** partially separated untreated allogeneic from syngeneic samples across days 7 and 14 (n = 4–5/group/time point), but **(b)** did not distinguish rejection (n = 2–3/time point) from acceptance (n = 13–14/time point) in anti-CD40L-treated allogeneic recipients across days 7 and 14.
